# [Corrigendum] MicroRNA-101 suppresses progression of lung cancer through the PTEN/AKT signaling pathway by targeting DNA methyltransferase 3A

**DOI:** 10.3892/ol.2026.15825

**Published:** 2026-08-20

**Authors:** Lumin Wang, Jiayi Yao, Hongfei Sun, Kang He, Dongdong Tong, Tusheng Song, Chen Huang

Oncol Lett 13: 329–338, 2017; DOI: 10.3892/ol.2016.5423

Subsequently to the publication of the above article, the authors have realized that Figs. 3 and 6 in their paper were assembled incorrectly; specifically, for the western blots shown in Fig. 3H on p. 333, the data were incorrectly chosen to represent the cyclin A2, AKT and p-AKT data, and the AKT data were also chosen incorrectly for Fig. 3D. As far as Fig. 6 was concerned, for Fig. 6E on p. 336, the wrong set of β-actin bands were chosen.

Subsequently, an interested reader drew to the authors’ attention that the tumour sizes reported at the end of the experiment (day 30) in the barchart in Fig. 6B were almost three-fold smaller than the sizes calculable from the tumours shown in Fig. 6A. In their response, the authors explained that the data from two mice which died during the course of the experiment were erroneously factored into the calculation (the authors offered a further explanation that the deaths of these mice may have been attributable to individual variations in sensitivity to the *in vivo* assay, including metabolic differences, inflammatory responses and undetectable tumor micrometastases), and also that the formula used for calculating the tumour sizes (V=L*W2/2) was applied incorrectly. Consequently, Fig. 6 was further revised to reflect the true sizes of the tumours in Fig. 6B.

The corrected versions of Figs. 3 and 6 are shown on the next two pages. Note that the revisions made to these figures do not affect the overall conclusions reported in the paper. The authors are grateful to the Editor of *Oncology Letters* for allowing them the opportunity to publish this Corrigendum, and all authors agree with its publication. Furthermore, the authors apologize to the readership for any inconvenience caused.

## Figures and Tables

**Figure 3.**
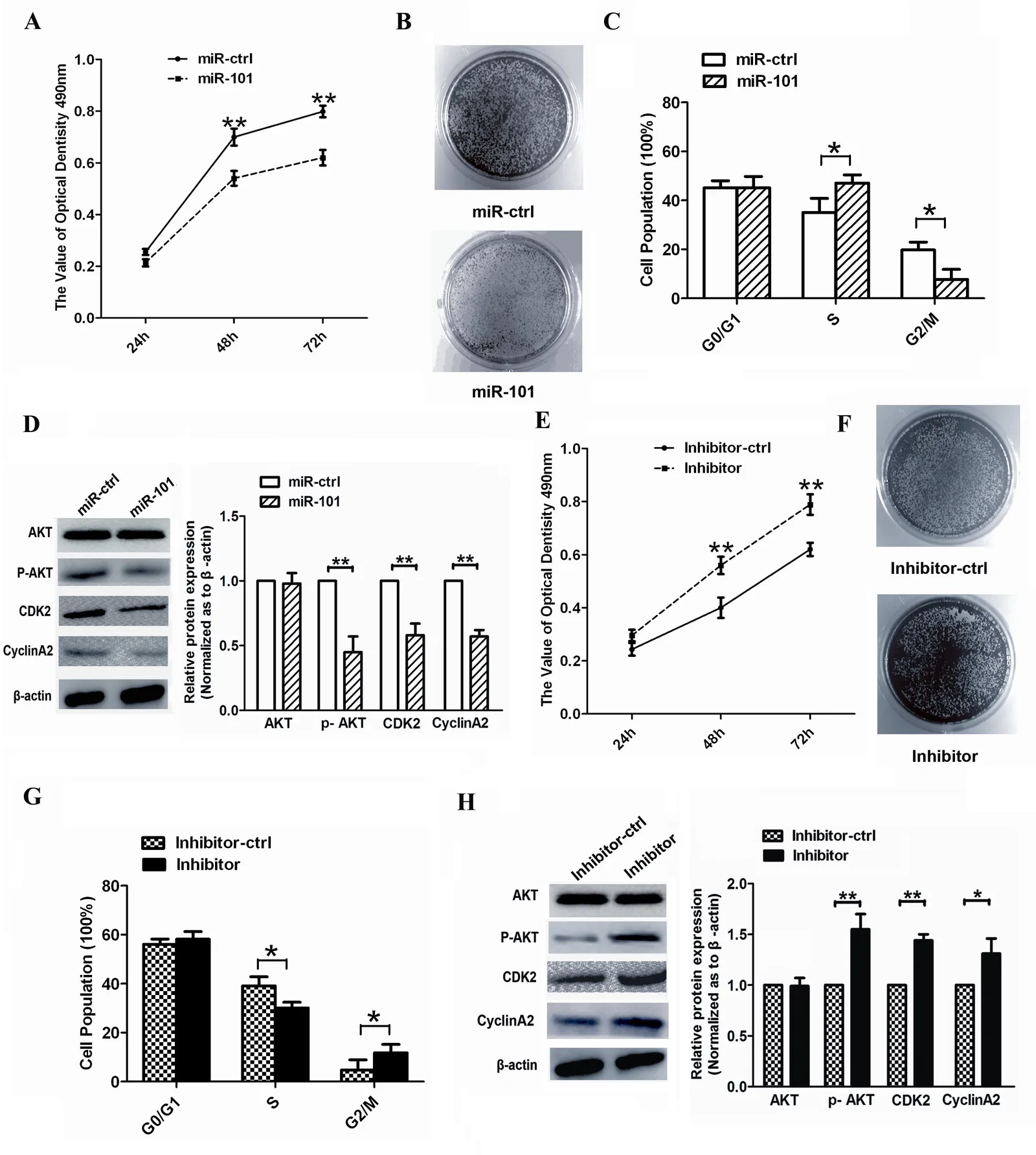
miR-101 decreases A549 cell proliferation and induces S-phase arrest *in vitro.* (A) The effects of miR-101 on A549 cell proliferation were determined by MTT assay at 24, 48 and 72 h. (B) Representative results of colony formation of A549 cells following miR-101 overexpression. (C) The cell cycle was determined in A549 cells 48 h after transfection. (D) Analysis of the expression of S-phase regulatory protein in A549 cells following transfection with miR-ctrl and miR-101 overexpression construct. (E) MTT was used to assay the effects of miR-101 inhibitor on the proliferation of A549 cells. (F) The growth of A549 cells was detected by colony formation following transfection of inhibitor. (G) The cell cycle was determined by transfecting with inhibitor or its control. (H) Expression of cell cycle regulators 48 h after transfection with inhibitor and control (*P<0.05, **P<0.01, Student's t-test).

**Figure 6.**
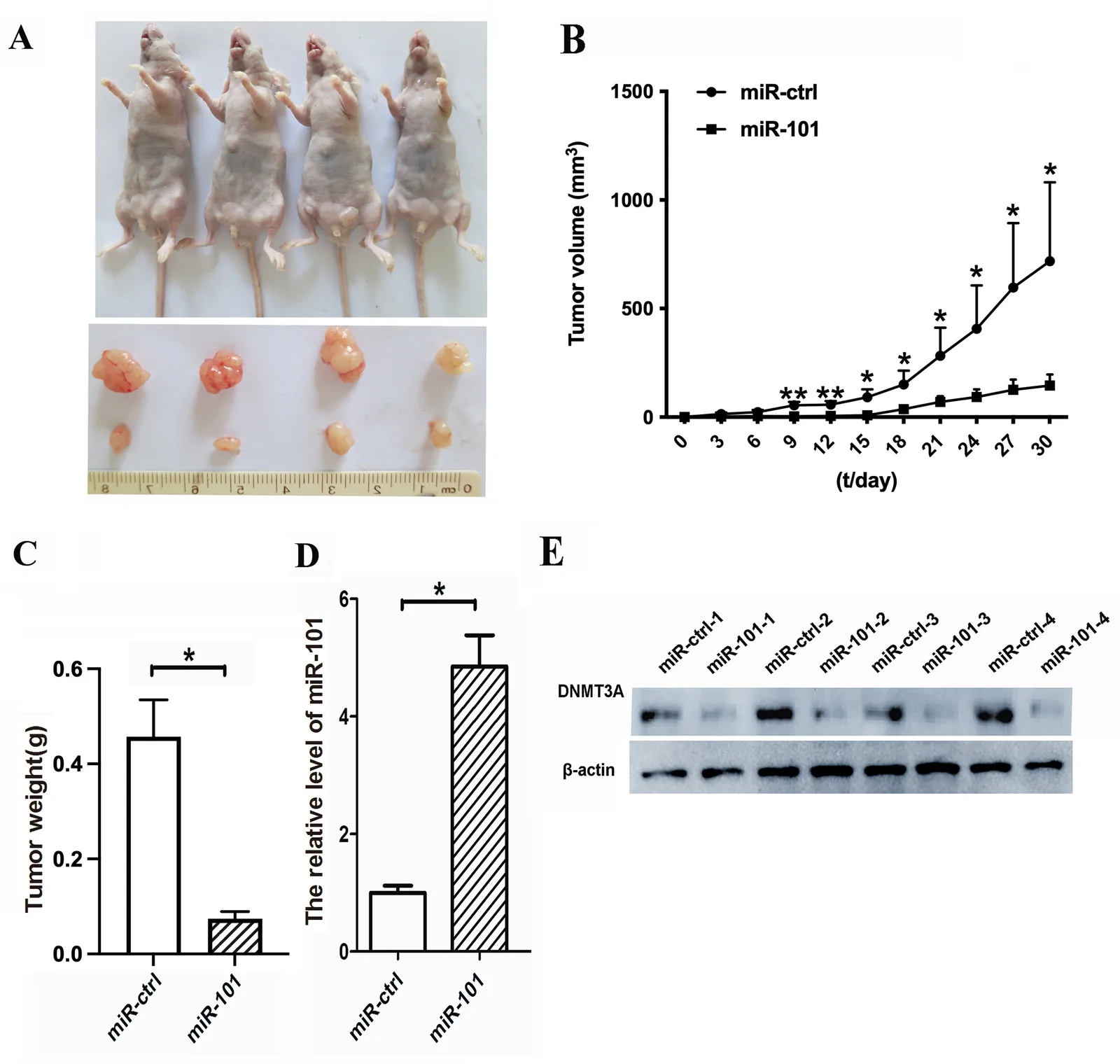
miR-101 inhibits lung cancer progression *in vivo.* (A) Small animal imaging analysis was used to assess the tumor volume *in situ* during the fourth week of tumor development. The flanks of four nude mice were injected with lentiviral vector (LV)-miR-101-transfected cells (left flank) and LV control (right flank), respectively. The lower portion reveals the morphology of mice injected with miR-101 and miR-ctrl. (B) Tumor growth curves. (C) Tumor weight. (D) Expression levels of miR-101 were measured by reverse transcription-quantitative polymerase chain reaction analysis in the tumor tissues from the animals. (E) Expression levels of DNA methyltransferase 3A (DNMT3A) were assessed by western blot analysis in tissues from the animals. β-actin was used as a housekeeping control (*P<0.05, **P<0.01, Student's t-test).

